# Interpretable AI for treatment decision-making in immunoradiotherapy of locally advanced nasopharyngeal carcinoma

**DOI:** 10.3389/fonc.2026.1775802

**Published:** 2026-03-06

**Authors:** Guili Cao, Bin Zeng, Zifu Yuan, Xiao Hu, Hai Ou

**Affiliations:** 1 Department of Oncology, First People’s Hospital of Zigong, Zigong Medica Science Academy, Zigong, China; 2 Department of Oncology, Jianyang People’s Hospital, Jianyang, China; 3 Department of Oncology, Yangjiang People’s Hospital, Yangjiang, China

**Keywords:** artificial intelligence, chemotherapy, immunotherapy, nasopharyngeal carcinoma, radiotherapy

## Abstract

**Background:**

Survival remains heterogeneous in locally advancednasopharyngeal carcinoma (NPC) despite immunotherapy, highlighting the need for explainable artificial intelligence (AI) for risk-adapted care.

**Methods:**

We retrospectively analyzed 249 patients with locally advanced NPC between 2018 and 2025. Patients were randomly split into a training cohort (70%) and a validation cohort (30%). A Cox–XGBoost survival modeling framework was developed using routinely available clinical variables to generate individualized risk scores and classify patients into low- and high-risk groups. Model discrimination was assessed using time-dependent ROC analysis. SHAP (SHapley Additive exPlanations) was applied to provide transparent, feature-level and patient-level interpretations of predicted risk.

**Results:**

Univariable Cox regression identified age, tumor grade, and N stage as significant prognostic factors. In the training cohort, the XGBoost-derived risk score robustly separated low- and high-risk groups, with significantly prolonged survival in the low-risk group (P < 0.001). In the validation cohort, the AUCs for predicting 1-, 2-, and 3-year OS were 0.784, 0.765, and 0.725, respectively. SHAP analyses consistently highlighted age as the strongest driver of predicted risk, followed by N stage and tumor grade; older age and advanced nodal disease were associated with higher predicted mortality risk.

**Conclusion:**

An interpretable XGBoost-based survival model built from routine clinical variables provides clinically meaningful risk stratification for locally advanced NPC patients.

## Background

Nasopharyngeal carcinoma (NPC) is a malignancy with a distinct geographic distribution and a high incidence in Southeast Asia (1). Its epidemiology and clinical behavior vary substantially across age groups and are influenced by sex and genetic susceptibility (2). For patients with locally advanced disease, definitive radiotherapy combined with chemotherapy remains the therapeutic backbone (3). Despite improved locoregional control, a non-negligible proportion of patients still experience recurrence or progression, and the burden of treatment-related toxicities can be considerable (4). Accordingly, there is a persistent need to refine multimodal strategies and deliver therapy in a more individualized manner.

The rapid development of immunotherapy—particularly immune checkpoint inhibition—has expanded treatment options and reshaped paradigms in several cancers (5, 6). The phase III CONTINUUM (7) trial evaluated the addition of sintilimab to chemoradiotherapy, with event-free survival (EFS) as the primary endpoint. At a median follow-up of 42 months, sintilimab significantly improved EFS versus standard therapy, with 36-month EFS rates of 86% vs 76% (HR, 0.59; 95% CI, 0.38–0.92; P = 0.019). However, response and survival benefits remain variable, reflecting underlying tumor heterogeneity and host-related determinants. Emerging evidence suggests that age may serve as a key modifier of outcome in the immunotherapy era, with younger patients often achieving more favorable survival under immuno-chemoradiotherapy (8). These observations indicate that age may capture clinically relevant differences in immune competence, comorbidity burden, and treatment tolerance, but age alone cannot fully guide personalized treatment decisions.

In parallel, artificial intelligence (AI)—particularly machine learning—has accelerated progress in prognostic modeling by leveraging routinely collected clinical datasets to uncover non-linear relationships that traditional methods may not capture (9). XGBoost (Extreme Gradient Boosting) is a widely used algorithm recognized for strong performance in complex, high-dimensional settings and has shown utility in oncology for risk stratification and outcome prediction (10–13).

Against this backdrop, we conducted a multicenter retrospective study to develop an interpretable AI model for survival prediction in locally advanced NPC treated with immuno-chemoradiotherapy. Our objective was to generate an explainable risk score that can support precision immunoradiotherapy-based decision-making, including risk-adapted treatment planning and follow-up.

## Methods

### Patient selection

Between 2018 and 2025, we retrospectively identified 249 consecutive patients with locally advanced NPC from three participating hospitals. Tumor staging was determined according to the American Joint Committee on Cancer/Union for International Cancer Control (AJCC/UICC) TNM staging system, 8th edition.

All data were de-identified prior to analysis. Given the retrospective nature of the study and the use of anonymized clinical information, the Ethics Committee of the First People’s Hospital of Zigong waived the requirement for additional ethical review for this secondary analysis, and written informed consent had been obtained from patients at the time of initial treatment. The study was conducted in accordance with the Declaration of Helsinki.

Inclusion criteria were:

1. Histologically confirmed NPC.
2. Clinical stage T3–4 N1–3 M0.
3. Complete baseline clinical, laboratory, and imaging data available.
4. Age ≥18 years.

Exclusion criteria included:

1. Distant metastasis at initial diagnosis.
2. Prior or synchronous malignancies other than NPC.
3. Incomplete treatment, missing key variables, or inadequate follow-up.
4. Severe uncontrolled comorbidities likely to independently impact survival.

### Treatment

All patients underwent definitive chemoradiotherapy; immunotherapy was administered in a subset of patients according to physician discretion and clinical indications. The prescribed dose was 66–72 Gy delivered in 2 Gy fractions, five days per week, over approximately 6–7 weeks. Intensity-modulated radiotherapy (IMRT) was used to treat the primary tumor and regional lymphatic drainage areas, with target volumes individualized to tumor extent and anatomy to optimize tumor control while limiting normal tissue exposure.

Concurrent platinum-based chemotherapy (cisplatin or carboplatin) was administered during radiotherapy. Adjuvant chemotherapy was delivered at the treating physician’s discretion, typically 2–4 cycles after radiotherapy.

### AI model development and interpretability

Univariable Cox regression was initially performed to identify baseline variables associated with OS, including sex, age, tumor grade, T stage, and N stage. Patients were randomly assigned to a training cohort and a validation cohort in a 7:3 ratio. Using the training cohort, we built an XGBoost model within a Cox survival learning framework to generate an individualized risk score for each patient. Patients were stratified into low- and high-risk groups using the median model-derived risk score as the cutoff.

Model performance was evaluated in the validation cohort using time-dependent ROC analysis. To enhance transparency, SHAP was used to quantify feature contributions and visualize both global feature importance and patient-level drivers of predicted risk.

### Statistical analysis

Continuous variables were compared using the Student’s t-test (or Mann–Whitney U test when non-normally distributed), and categorical variables were compared using the Chi-square test. OS was estimated using the Kaplan–Meier method, and survival differences were assessed with the log-rank test. Statistical analyses were performed in R software. A two-sided p-value <0.05 was considered statistically significant.

## Results

### Baseline characteristics

A total of 249 patients with locally advanced NPC were included. Overall, 186 (74.7%) were male and 63 (25.3%) were female, with a mean age of 56.2 ± 13.3 years. Most patients had grade III/IV tumors (76.3%), T4 disease (53.8%), and N2 nodal status (52.2%), followed by N1 (34.9%) and N3 (12.9%).

Patients were randomly assigned to a training cohort and a validation cohort. Baseline distributions of sex, age, histologic grade, T stage, and N stage were comparable between cohorts (all p > 0.05), indicating adequate balance. Baseline clinicopathologic characteristics were also similar between patients aged <60 years and those aged ≥60 years (all p > 0.05, **Table 1**).

**Table 1:** Baseline characteristics of the overall cohort, stratified by age group and dataset.

Variable	ALL	Age ≥60		p	Test	Train	p
Total	249	153	96		80	169	
Sex				0.717			0.936
Female	63 (25.3%)	37 (24.2%)	26 (27.1%)		21 (26.2%)	42 (24.9%)	
Male	186 (74.7%)	116 (75.8%)	70 (72.9%)		59 (73.8%)	127 (75.1%)	
Age, mean ± SD	56.2 (13.3)				58.6 (14.1)	55.1 (12.8)	0.064
Grade				0.399			1
I/II	59 (23.7%)	33 (21.6%)	26 (27.1%)		19 (23.8%)	40 (23.7%)	
III/IV	190 (76.3%)	120 (78.4%)	70 (72.9%)		61 (76.2%)	129 (76.3%)	
T				0.631			0.673
T3	115 (46.2%)	73 (47.7%)	42 (43.8%)		39 (48.8%)	76 (45.0%)	
T4	134 (53.8%)	80 (52.3%)	54 (56.2%)		41 (51.2%)	93 (55.0%)	
N				0.055			0.123
N1	87 (34.9%)	45 (29.4%)	42 (43.8%)		26 (32.5%)	61 (36.1%)	
N2	130 (52.2%)	85 (55.6%)	45 (46.9%)		48 (60.0%)	82 (48.5%)	
N3	32 (12.9%)	23 (15.0%)	9 (9.38%)		6 (7.5%)	26 (15.4%)	

### Univariable Cox analysis and overall survival

Univariable Cox regression identified age, tumor grade, and N stage as significant prognostic factors for OS, while sex and T stage were not significantly associated with survival (**Table 2**).

**Table 2:** Univariable Cox regression analysis of clinical variables.

Variable	Category	HR (95% CI), p
Sex	Female	Reference
	Male	0.87 (0.61–1.23), p = 0.429
Age	Continuous	1.01 (1.00–1.03), p = 0.016
Grade	I/II	Reference
	III/IV	0.65 (0.46–0.91), p = 0.011
T stage	T3	Reference
	T4	1.06 (0.78–1.44), p = 0.700
N stage	N1	Reference
	N2	1.13 (0.81–1.57), p = 0.472
	N3	1.66 (1.04–2.66), p = 0.033

**Figure 1:**
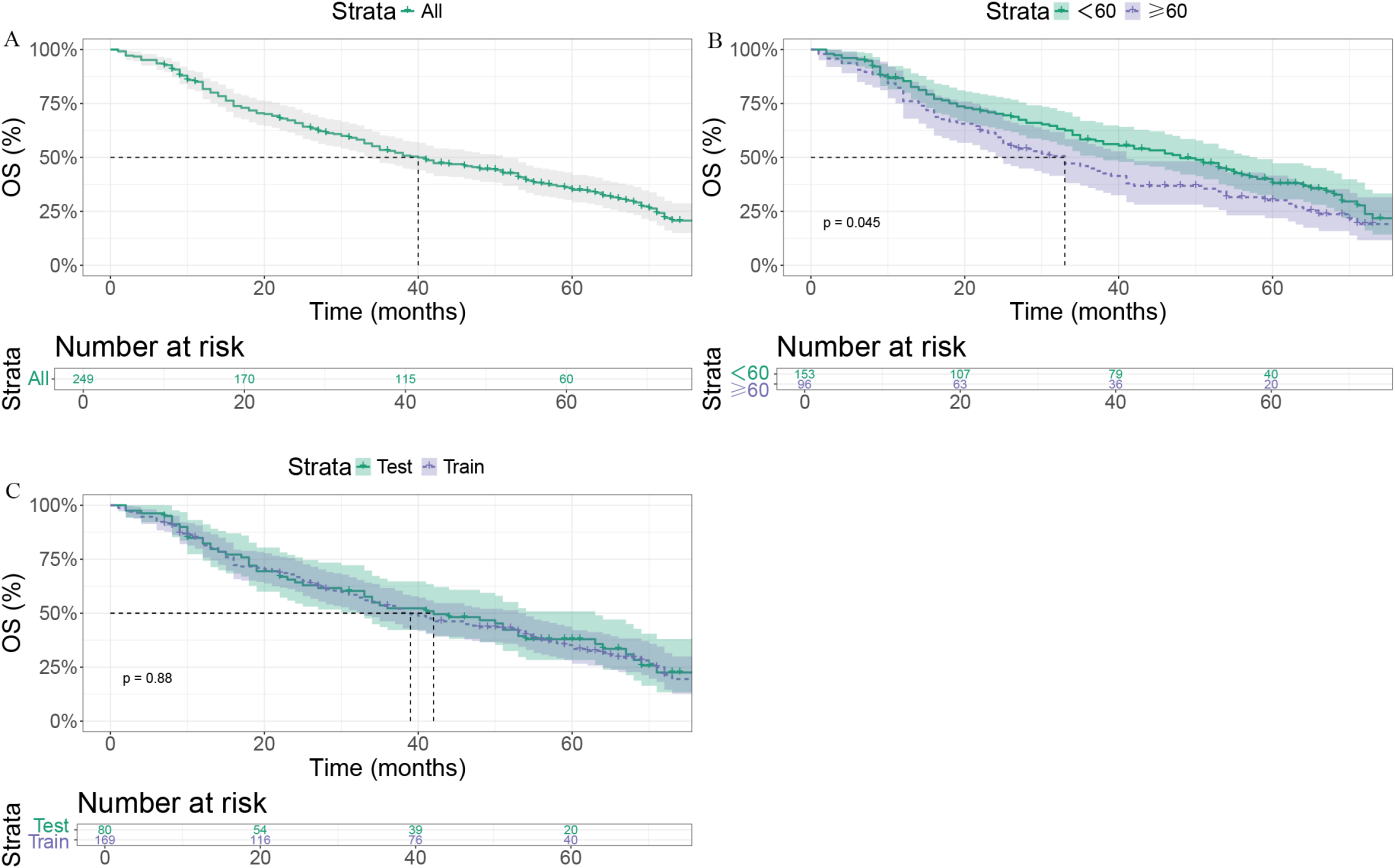
Kaplan–Meier survival curves. **(A)** Overall survival (OS) for all patients. **(B)** OS stratified by age (<60 vs. ≥60 years), showing poorer survival in older patients (p = 0.045). **(C)** OS comparison between the training and test cohorts, with no significant difference (log-rank p = 0.88).

In the entire cohort, median OS (mOS) was 40 months (95% CI: 34–51 months, **Figure 1A**). Patients aged <60 years had significantly longer OS than those aged ≥60 years (48 vs. 33 months; p = 0.045, **Figure 1B**). Median OS was 39 months (95% CI: 33–53 months) in the training cohort and 42 months (95% CI: 33–63 months) in the validation cohort, without a significant difference (P = 0.88, **Figure 1C**).

### Cox–XGBoost survival model

**Figure 2:**
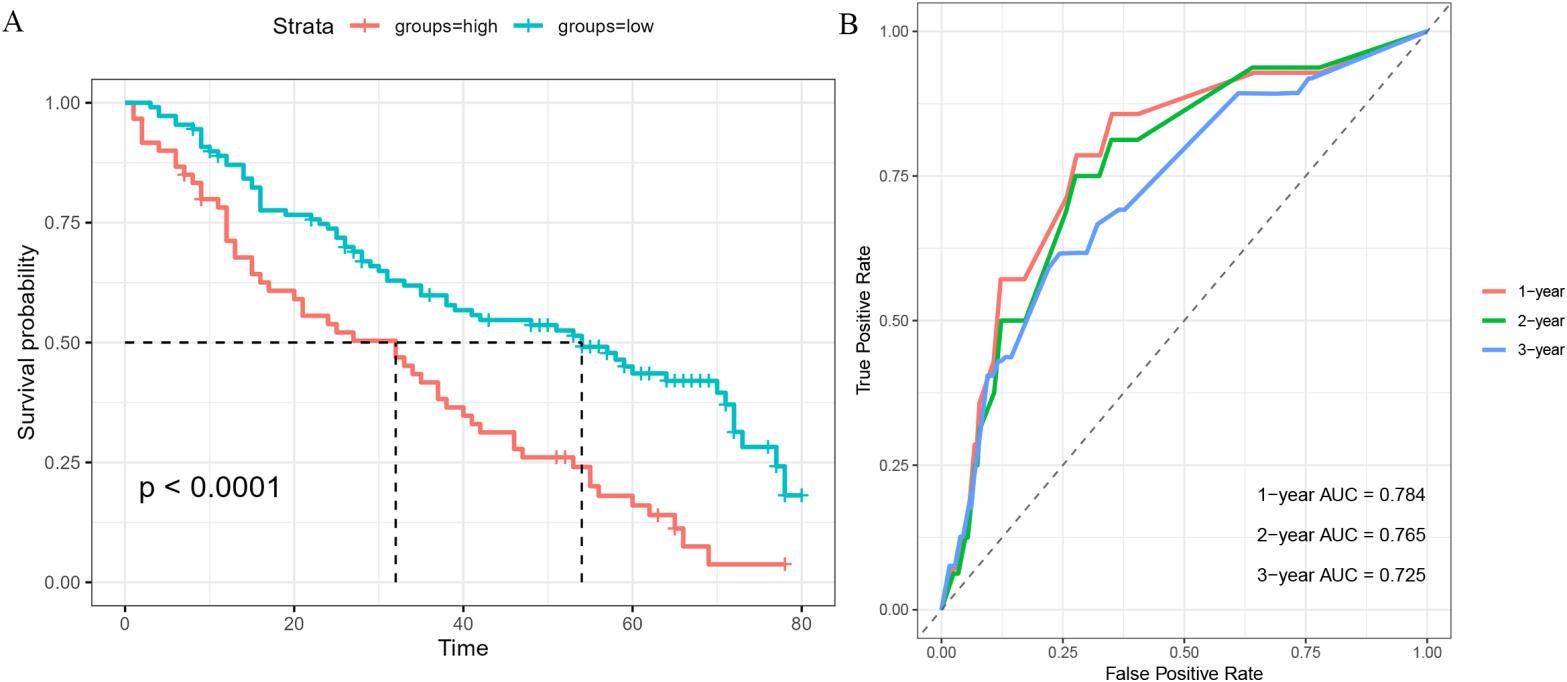
Model performance for risk stratification. **(A)** Kaplan–Meier survival curves for high- and low-risk groups. **(B)** Time-dependent ROC curves for 1-, 2-, and 3-year overall survival prediction.

Using the training cohort, we developed an XGBoost survival model incorporating age, tumor grade, and N stage to compute individualized risk scores. Patients were dichotomized into high- and low-risk groups based on the median risk score. Kaplan–Meier curves demonstrated significantly prolonged OS in the low-risk group compared with the high-risk group (P < 0.001, **Figure 2A**). In the validation cohort, time-dependent ROC analysis demonstrated good discriminatory performance, with AUCs of 0.784, 0.765, and 0.725 for 1-, 2-, and 3-year OS, respectively (**Figure 2B**).

**Figure 3:**
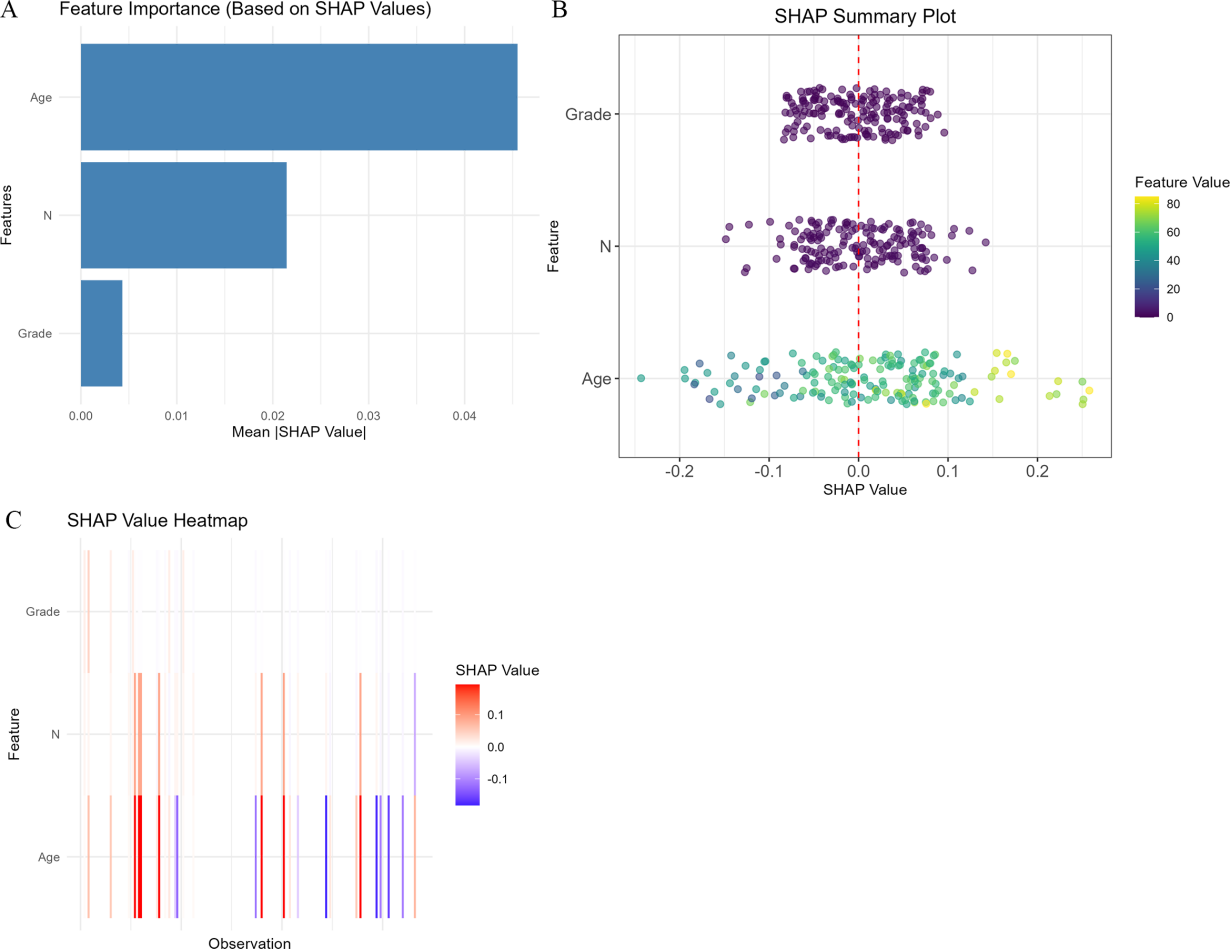
SHAP interpretation of the Cox–XGBoost model. **(A)** Feature importance ranking, with age as the most influential factor, followed by N stage and grade. **(B)** SHAP summary plot showing that higher age, advanced N stage, and higher grade are associated with increased risk. **(C)** SHAP heatmap illustrating patient-level feature contributions.

To interpret the model, SHAP analyses were performed. The feature-importance plot (**Figure 3A**) ranked age as the leading contributor to risk prediction, followed by N stage and tumor grade. The SHAP summary plot (**Figure 3B**) showed that higher age generally corresponded to higher predicted risk (positive SHAP values), and advanced N stage and higher tumor grade similarly shifted predictions toward higher risk. The SHAP heatmap (**Figure 3C**) provided patient-level visualization, with clusters of positive SHAP values more frequently observed among older patients and those with advanced nodal disease.

**Figure 4:**
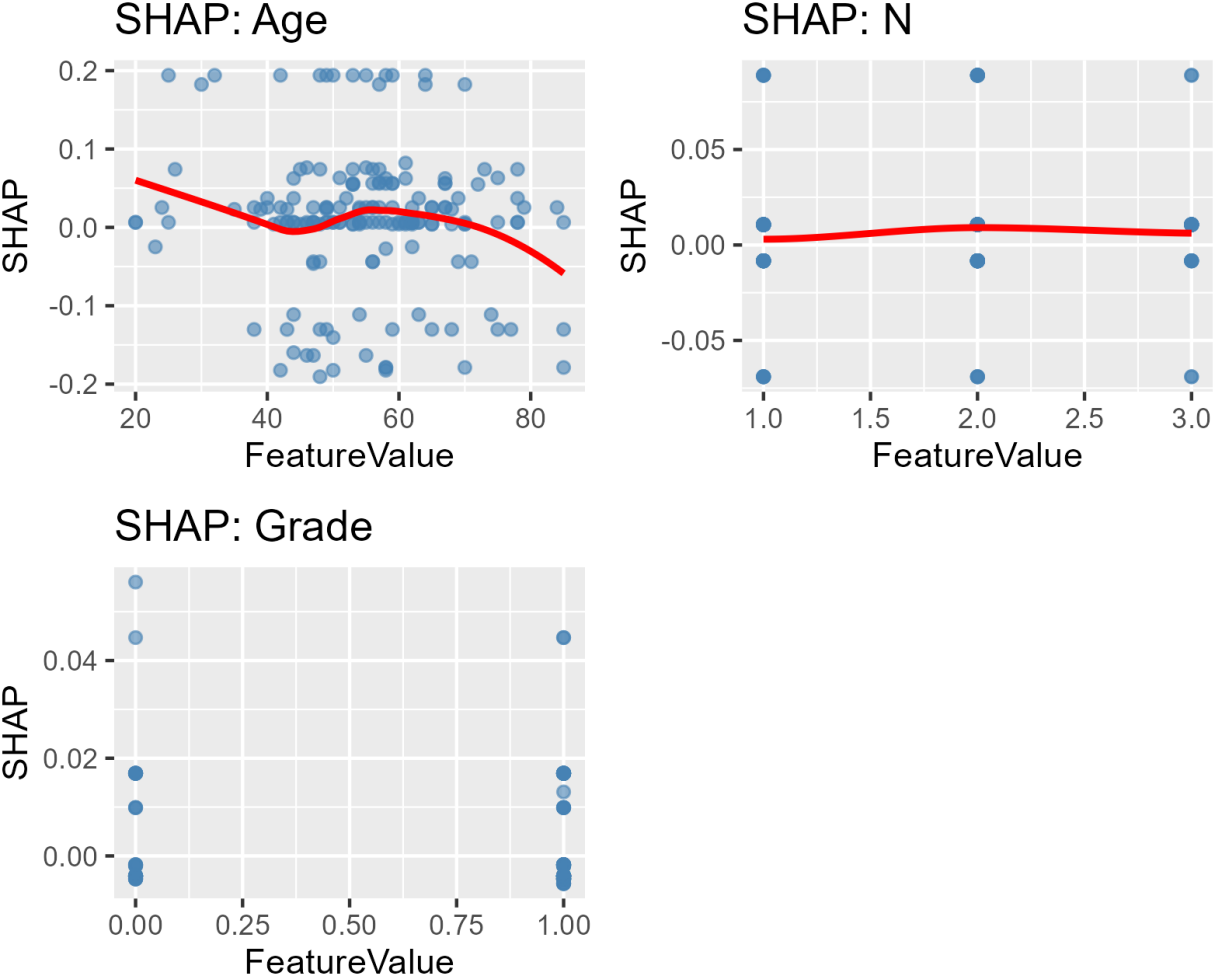
SHAP dependence plots for key variables. Dependence plots for age, N stage, and grade, demonstrating their marginal effects on predicted risk.

SHAP dependence plots for age, N stage, and tumor grade are shown in **Figure 4**. Age demonstrated a mild non-linear association with predicted risk, with a modest risk increase between 50 and 60 years. N stage showed a moderate effect, while tumor grade clearly separated lower- from higher-risk predictions.

## Discussion

NPC is highly prevalent in Southeast Asia, with southern China representing a well-recognized high-incidence region (14). Although chemoradiotherapy remains standard for locally advanced disease, its effectiveness may be limited by recurrence risk and treatment-related toxicity (15). Immune checkpoint inhibitors have broadened therapeutic options for NPC and demonstrated encouraging activity (16). However, the heterogeneity of outcomes under immuno-chemoradiotherapy creates a practical need for tools that can guide patient selection, risk-adapted management, and follow-up intensity.

Immunotherapy has become an increasingly important component of NPC management, supported by multiple phase III trials demonstrating clinically meaningful benefit with PD-1 blockade. In recurrent/metastatic NPC, CAPTAIN-1st (17) showed that camrelizumab plus gemcitabine–cisplatin (GP) significantly prolonged progression-free survival compared with placebo plus GP (median PFS, 9.7 vs 6.9 months; HR 0.54; P = 0.0002). In the first-line setting, JUPITER-02 (18) demonstrated that toripalimab plus gemcitabine/cisplatin significantly improved both PFS (median PFS, 21.4 vs 8.2 months; HR 0.52) and overall survival (median OS, NR vs 33.7 months; HR 0.63; P = 0.008) versus placebo plus chemotherapy. RATIONALE-309 (19) further confirmed a significant PFS benefit with tislelizumab plus chemotherapy compared with placebo plus chemotherapy (HR 0.52, 95% CI 0.38–0.73; P < 0.0001). Collectively, these phase III data establish the therapeutic value of PD-1 blockade in NPC and support its broader integration and ongoing evaluation in multimodal strategies, including curative-intent treatment for locoregionally advanced disease. Nevertheless, outcomes remain heterogeneous in real-world practice, underscoring the need for practical and explainable tools for risk stratification to guide individualized management.

In this multicenter retrospective study, we developed and validated an interpretable AI-driven survival prediction model for locally advanced NPC treated with immuno-CRT. By integrating routinely available clinical factors—age, tumor grade, and N stage—we generated individualized risk scores that stratified patients into clinically distinct prognostic groups. SHAP explanations provided transparent insights into how each variable contributed to the predicted risk, supporting potential clinical adoption of the model as a decision-support aid.

Consistent with prior evidence, univariable Cox analyses identified age, tumor grade, and N stage as significant predictors of OS (20, 21). Higher tumor grade, reflecting more aggressive histologic behavior, was associated with worse outcomes, in keeping with established observations (22). T stage did not reach statistical significance in our analysis, which may reflect the relatively restricted T-stage range (T3–4) within a locally advanced cohort and the potentially greater prognostic weight of nodal burden for survival endpoints (23). Age emerged as the dominant driver of predicted risk, which is clinically plausible in the immunochemoradiotherapy era. Older patients may have immune senescence with weaker antitumor and EBV-specific immune responses, a higher comorbidity burden and competing mortality risk, and reduced treatment tolerance that can lead to dose reductions or interruptions (20). Notably, the SHAP dependence plot suggested that the age–risk association was not strictly linear, with risk increasing more prominently at older ages, supporting flexible modeling and individualized risk assessment.

XGBoost was selected because it can capture non-linear effects and potential interactions without prespecifying functional forms, and it is robust to heterogeneity via ensemble learning and regularization. Importantly, SHAP provides transparent global and patient-level explanations, which is essential for applying the model as a practical risk-stratification tool in real-world NPC care (24). In the training cohort, the model clearly separated low- and high-risk groups, suggesting that an AI-derived risk score may support risk-adapted clinical strategies—such as closer surveillance, proactive toxicity monitoring, and individualized supportive care—within institutional treatment protocols. Validation cohort AUCs for 1–3 years further support clinically meaningful predictive value in the short-to-intermediate term.

Interpretability is critical for deploying AI tools in routine oncology workflows. The SHAP analyses in our study consistently identified age as the dominant driver of predicted mortality risk, followed by N stage and tumor grade. This finding may reflect the composite impact of age-related immune function, comorbidity burden, and treatment tolerance, which can influence both the effectiveness and feasibility of immuno-CRT. At the patient level, SHAP heatmaps and dependence plots illustrated how combinations of older age and advanced nodal disease contributed to higher predicted risk, providing actionable transparency for clinicians (25–29). Collectively, these results support the clinical premise that explainable AI may help operationalize precision immunoradiotherapy-based management.

Compared with a conventional Cox model, which assumes a linear association between covariates and the log hazard unless non-linear terms or interactions are explicitly specified, the Cox–XGBoost framework can learn non-linear effects and potential interactions directly from the data by optimizing the Cox partial-likelihood within gradient boosting (30). Importantly, the model outputs an interpretable relative risk score, enabling clinically actionable risk stratification. From a practical standpoint, patients predicted to be high-risk may require more cautious benefit–risk evaluation, closer monitoring of treatment tolerance and toxicities, and more intensive follow-up and supportive care, even within immunotherapy-containing multimodal strategies.

Several limitations warrant consideration. First, the retrospective design introduces potential selection bias and residual confounding. Second, although this was a multicenter study, the model was developed and validated within the participating centers and therefore lacks true external validation in independent cohorts outside these centers, which may restrict generalizability across institutions and treatment protocols. Future work should include prospective validation and/or external multicenter validation by applying the locked model to independent cohorts with standardized baseline data collection, treatment definitions, and follow-up schedules, and assessing discrimination and calibration with recalibration if needed. Third, while XGBoost performed well, other algorithms (e.g., random forests or deep learning approaches) may yield additional gains and should be explored. Finally, our model relied on clinical variables only; integrating radiomics, genomic/transcriptomic, or immune biomarkers could strengthen precision and biological interpretability (31, 32).

## Conclusion

This study demonstrates that an interpretable XGBoost-based AI model can provide clinically useful survival prediction and risk stratification for patients with locally advanced NPC undergoing immuno-chemoradiotherapy. Using readily available clinical variables, the model offers transparent, patient-level explanations and may support precision immunoradiotherapy-based treatment planning and individualized follow-up in real-world practice.

## Data Availability

The raw data supporting the conclusions of this article will be made available by the authors, without undue reservation.
